# Randomized Controlled Trial of the Clinical Recovery and Biodegradation of Polylactide-co-glycolide Implants Used in the Intramedullary Nailing of Children’s Forearm Shaft Fractures with at Least Four Years of Follow-Up

**DOI:** 10.3390/jcm10050995

**Published:** 2021-03-02

**Authors:** Marja Perhomaa, Tytti Pokka, Linda Korhonen, Antti Kyrö, Jaakko Niinimäki, Willy Serlo, Juha-Jaakko Sinikumpu

**Affiliations:** 1 Research Unit of Medical Imaging, Physics and Technology (MIPT), Department of Diagnostic Imaging, Oulu University Hospital, PoB 50, 90029 Oulu, Finland; jaakko.niinimaki@ppshp.fi; 2 Department of Children and Adolescents/Pediatric Surgery and Orthopedics, PEDEGO Research Center, Oulu University Hospital, 90029 Oulu, Finland; tytti.pokka@ppshp.fi (T.P.); linda.korhonen@lshp.fi (L.K.); willy.serlo@ppshp.fi (W.S.); juha-jaakko.sinikumpu@ppshp.fi (J.-J.S.); 3 Department of Children, Pediatric Surgery, Päijät-Häme Central Hospital, FIN-Keskussairaalankatu 7, 15850 Lahti, Finland; antti.kyro@fimnet.fi

**Keywords:** biodegradable fracture fixation, forearm fracture, paediatric, long-term outcomes, magnetic resonance imaging (MRI)

## Abstract

The preferred surgical fixation of forearm shaft fractures in children is Elastic Stable Intramedullary Nailing (ESIN). Due to known disadvantageous effects of metal implants, a new surgical method using biodegradable polylactide-co-glycolide (PLGA) intramedullary nails has been developed but its long-term outcomes are unclear. The aim of this study was to compare the long-term outcomes of Biodegradable Intramedullary Nailing (BIN) to ESIN and assess the biodegradation of the study implants via magnetic resonance imaging (MRI). The study population of the prospective, randomized trial consisted of paediatric patients whose forearm shaft fractures were treated with BIN (*n* = 19) or ESIN (*n* = 16). Forearm rotation at minimally four years’ follow-up was the main outcome. There was no clinically significant difference in the recovery of the patients treated with the BIN as compared to those treated with the ESIN. More than half of the implants (57.7%, *n* = 15/26) were completely degraded, and the rest were degraded almost completely. The PLGA intramedullary nails used in the treatment of forearm shaft fractures in this study resulted in good function and anatomy. No unexpected disadvantages were found in the degradation of the implants. However, two implant failures had occurred in three months postoperatively.

## 1. Introduction

In traumatology, bioabsorbable implants have been used in different indications for several decades with promising outcomes [1,2,3,4,5,6,7,8,9]. The traditional fixation methods have some disadvantages that the techniques using biomaterials and their distinct features aim to overcome. The degradation of biodegradable polymers occurs mainly via hydrolysis and secondarily through non-specific enzymatic pathways. The degradation rate depends on the geometry and size of the implant as well as molecular characteristics, such as the ratio of different copolymers [3]. Contrary to conventional implant materials, such as titanium alloy, biodegradable implants are not inert in the human body and foreign-body reaction is evident [2,3]. This inflammatory tissue response is in most cases clinically not important [2]. However, osteolysis with biodegradable devises is considered to be the most common adverse event of these implants in orthopaedic practice [10,11].

Forearm fractures are common in the paediatric population, accounting for up to 36% of all fractures [12]. In most cases, these fractures can be treated via closed reduction and plaster cast immobilization with good outcomes. Diaphyseal forearm fractures have less remodelling potential than metaphyseal fractures; thus, the limitations of acceptable alignment are lower than for distal metaphyseal or physeal fractures [13]. With paediatric diaphyseal forearm fractures, operative treatment should be reserved for patients whose fracture alignment cannot be achieved through closed means [14]. Hence, irreducible fractures or fractures that fail to maintain satisfactory reduction require osteosynthesis to achieve healing without producing anatomical or functional abnormalities [15,16]. In addition, severe open fractures and fractures with neurovascular compromising or compartment syndrome are indications for operative treatment. Schmittenbecher offers a treatment algorithm for forearm shaft fractures in children in which unstable or irreducible fractures and fractures with final axial deviation >10° should be treated operatively [15]. He encourages to manage every patient by a primary definitive method to avoid more than one surgical intervention. Complete shaft fracture of both bones on the same level, with oblique fracture lines and convergent displacement, is classified as unstable forearm shaft fracture. The predicting factors of conservative treatment failure include casting index ≥0.84 [17], translational dislocation ≥10 mm, older age and male gender [18]. It has been reported that the operative treatment of these fractures has become more frequent in developed countries [14,19].

Elastic stable intramedullary nailing (ESIN) is currently the recommended operative treatment of forearm shaft fractures in children [14,20,21,22,23,24,25,26,27,28]. ESIN fulfils all the criteria of minimally invasive bone surgery: shorter operating time, minimal soft-tissue dissection, smaller incisions and thus smaller scars, less pain, and earlier mobilization [23]. ESIN is an ideal procedure for a transverse diaphyseal forearm fractures in children and when indications are correct and biomechanical principles are respected during application, rate of complication is very low [27]. Alternatively, especially in pubertal teenagers, fixation with plates and screws is an accepted option [14]. After bone healing, the intramedullary nails are usually removed during a second operation. This has several disadvantages, such as anaesthesia- and surgery-related risks, and concern, discomfort, and costs to the patients, their families, and health care.

A novel procedure called biodegradable intramedullary nailing (BIN) has been described [29]. The method is based on polylactide-co-glycolide intramedullary nails (ActivaNail IM™) designed and manufactured for research purposes by Bioretec Ltd. (Tampere, Finland). It was invented to obviate the disadvantages of ESIN in forearm shaft fractures. The minimum of two years’ results of that pioneering RCT study with biodegradable intramedullary nailing have been published previously by Korhonen et al. [30]. However, none of the patients in that preliminary study showed complete degradation of their implants via magnetic resonance imaging (MRI). Hence, the final outcomes of the technique and the evolution of the implants used in these patients has remained unclear. Further study was warranted to get a more precise idea of radiographic and functional outcomes in a longer time frame.

In this study, we aimed to investigate the long-term functional and radiographic outcomes of the operative treatment of children’s forearm fractures with biodegradable intramedullary nails. The biodegradation process of biodegradable polylactide-co-glycolide (PLGA) intramedullary nails is still unknown. Thus, we evaluated radiographic degradation at least four years after the operation.

## 2. Materials and Methods

### 2.1. Study Protocol and Patients

This is a prospective, randomized, controlled, clinical trial performed in two centers in Finland, Oulu University Hospital, Oulu, Finland, and Central Hospital of Päijät-Häme, Lahti, Finland, between November 2011 and January 2020. Patient enrolment occurred between November 2011 and January 2015. All consecutive patients, aged between five and 15 years, who suffered from single or both bone forearm shaft fracture requiring surgical fixation were aimed to be invited. After operating 1–2 preliminary cases per study institute with the new study procedure, all eligible participants were randomized into two study groups for treatment either by BIN or ESIN. A research assistant, who was not involved in the operative treatment, was responsible for the randomization using varied block sizes to achieve 19 sealed envelopes per group. These envelopes were delivered to the study institutions upon request. When parents or the guardians signed the informed consent, a nurse at the ward not involved in the operative treatment of the patient opened one of these envelopes for the operating surgeon, who as part performed the procedure according to randomization [30].

Patients with open fractures, significant soft-tissue injury, pathological fractures or previous fracture or infection in the forearm were excluded. In addition, patients with metabolic bone diseases, systemic disease or medication affecting the bone quality, and resistance to infection or fractures older than seven days were excluded [30].

The study design and power analysis have been described in detail previously, when the short-term outcomes were concerned. It was calculated that 13 patients per group would be needed. To obviate the problems with possible drop-outs, an excess of minimum 20% per group was decided [30].

ESIN implants used in this study were Synthes TEN™ (DePuy Synthes, West Chester, PA, USA) implants. Their thickness was aimed to be 0.4 fold, compared to the minimum thickness of the intramedullary canal. ActivaNail IM™ biodegradable intramedullary nails were used in the BIN group, and the implants were produced by Bioretec Ltd, Tampere, Finland. There were three thickness of the nails available: 2.0 mm, 2.7 mm, and 3.2 mm. The PLGA nail is straight and without a curved tip. As great thickness as possible was selected, according to the diameter of the intramedullary canal; thickness was evaluated both with preoperative radiographs and by using the reamers intraoperatively. The hydrolytically activated memory effect of the implant material increases the nail diameter and decreases nail length by 1–2%, which may increase the stability of the fixation. The surgical technique of BIN was originally described in a comprehensive technical report in 2013 [29]. According to the study plan, all operated extremities were immobilized by using the above-the-elbow cast, regardless of the implants. The titanium ESIN implants were removed by default in every patient 4 to 6 months postoperatively.

**Figure 1:**
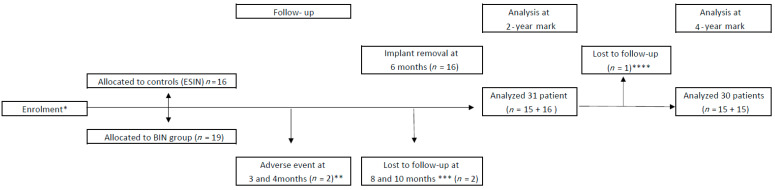
Flow chart of the randomized controlled trial in children with forearm shaft fractures treated either via bioabsorbable nailing (BIN) or elastic stable intramedullary nailing (ESIN). * The enrolment occurred between November 2011 and January 2015. ** Two patients suffered from implant failures. *** Two patients had high-energy injuries resulting in new forearm fractures. **** One patient was not attending because of a serious illness.

Altogether, 35 patients were first recruited: 19 were treated with BIN and 16 with ESIN. However, two cases treated with BIN suffered from new injuries resulting in re-fracture, and another two cases lost reduction unexpectedly [30] (Figure 1). One patient in the ESIN group did not complete the long-term follow-up due to them contracting a severe disease. Thus, the number of the patients at the final follow-up was 15 for both groups. A biodegradable nail was introduced to both forearm bones in 11 patients, to the ulna in three patients, and to the radius in one patient; the total number of bones with BIN fixation was 26. Thirteen out of 15 patients with ESIN were treated with both bone fixation and two had a nail in the radius only.

The clinical and radiographic long-term outcomes were investigated at an outpatient clinic visit at least four years after the operation between March 2019 and January 2020. The mean follow-up was 6.7 ± 1.2 years for BIN and 6.9 ± 1.0 for ESIN (*p* = 0.668). The mean age of the patients at follow-up was 17.2 ± 1.9 years in the BIN group and 17.1 ± 3.2 years in the ESIN group (*p* = 0.932). The male/female ratio was 5/10 for the BIN group and 7/8 for the ESIN group (*p* = 0.0082). The patient characteristics and injury types are presented in Table 1.

### 2.2. Clinical Investigation

The range of rotational movement in the forearm (degrees), wrist, and elbow movements, carrying angles and grip/pinch strength (Nm) were determined. Clinical investigation included Flynn’s criteria for anatomical and functional outcomes, the MAYO elbow performance score (0–100 points, with 100 points denoting the best possible performance), and QuickDASH (Disabilities of Arm, Shoulder and Hand Score with 0% denoting no disability, and 100% denoted most severe disability). Pain was recorded on a linear metric visual analogue scale (VAS, 0–100) in mm ± standard deviation (SD).

### 2.3. Imaging

Anterior-posterior and lateral radiographs of both forearms were taken. The radiographs were taken by means of digital radiography system on the bucky table with imaging distance of 115 cm. The images were obtained according to the imaging instructions of our institutes. The measurements were performed by paediatric radiologist with 20 years’ experience in paediatric imaging (MP). The intra-rater reliability was assessed [30], and it was found to be excellent, 0.941.

Tubularisation of the medulla, callus reabsorption, and visibility of the tri-calcium-phosphate (β-TCP) tip of the biodegradable nail were observed from the plain films. Diaphyseal angular deformity in both projections was measured. The length discrepancies between the fractured and uninjured forearm bones of each patient were assessed. The lengths of the radius and ulna were measured from the proximal end to the tip of the processus styloideus in both bones.

MRI of the fractured forearm in the BIN group was performed using Siemens Magnetom Espree 1.5 Tesla or Siemens Magnetom Avanto 1.5 Tesla (Siemens, Erlangen, Germany) equipment. The sequences were T1, STIR, and 3D T2 fat-saturated gradient echo in the sagittal plane and T1 and T2 in the axial plane. The resorption of the nail shaft, as well as the visibility of the nail canal, nail entrance, and β-TCP tip, was assessed. Intraosseal oedema, the presence of continuous fatty medullary bone, and the possible formation of intraosseal cysts were also detected. The interosseal membrane (IOM) between the radius and ulna was examined in axial sequences. Soft tissue oedema and operation-related artefacts were reported. The images were analysed by a paediatric radiologist with 20 years’ experience in paediatric imaging (MP).

### 2.4. Outcome Variables

The range of rotational movement in the forearm (degrees) was the main clinical outcome in this study. Secondary outcomes included wrist and elbow movements and grip/pinch strength, carrying angles, Flynn’s criteria, the MAYO elbow performance score and QuickDASH. Pain, being also a secondary outcome, was recorded by using a visual analogue scale (VAS). Secondary outcomes included imaging findings at the four-years mark: bone remodelling, degradation of the biodegradable nails, and their tri-calcium-phosphate (β-TCP) tips and other implant-related imaging findings.

### 2.5. Statistical Methods

For functional outcome, we did both per protocol (PP) and intention-to-treat (ITT) analysis. In ITT analysis, the last available functional results were used from the drop outs. Otherwise, the results included per protocol groups.

Differences in the functional outcomes between treatments were tested by Student’s *t*-test. Standardized Normal Deviate (SND) test was used to compare differences in proportions. 5% was considered to be the level of statistical significance (*p* < 0.05). All tests were two-sided, and 95% confidence intervals (CI) were used. Statistical analyses were performed by using IBM SPSS Statistics for Windows, Version 26 (IBM Corp., Armonk, NY, USA) and StatsDirect, Version 3 (StatsDirect Ltd., Cambridge, UK) statistical software.

### 2.6. Ethical Aspects

The study plan was originally approved by the Medical Ethics Committee and the Hospital Ethics Committee of Pirkanmaa Hospital District, Tampere, Finland (§R09231/2011) and recorded in the annals of the Northern Finland Hospital District, Oulu, Finland. The Medical Ethics Committee of Northern Finland Hospital District, Oulu, Finland approved the extension of the study (30 January 2019, 112/2018). Permission for the study was also approved by Päijät-Häme Central Hospital for the patients treated and re-examined at that hospital (6 November 2019, D/2219/13.00.00.00/2019). The Finnish National Supervisory Authority for Welfare and Health (Valvira, Helsinki, Finland) approved the implant in question for the study purpose in this research-intended study. This study was re-registered at ClinicalTrials.gov (NCT04385745), while the primary registration did not cover the extension of the study period. Written informed consent was obtained from eligible paediatric patients and/or their guardians or the adult patients themselves.

## 3. Results

### 3.1. Functional Results and Residual Symptoms

In PP analysis, the pronation of the forearm, flexion of the elbow, and volar flexion of the wrist were slightly better in the BIN patients than the ESIN patients, with the pronation being 79° (range 70°–90°) in the BIN group versus 73° (range 62°–88°) in the ESIN group (*p* = 0.030). Flexion of the elbow was 154° (range 140°–163°) for the BIN group and 145° (range 135°–153°) in the ESIN group (*p* = 0.001). The volar flexion of the wrist measured 89° (range 80°–100°) in the BIN group and 83° (range 67°–95°) in the ESIN group (*p* = 0.023). There were no significant differences between the groups in terms of the supination of the forearm, extension of the elbow, or dorsiflexion of the wrist. When the functions of the operated forearms were compared to the uninjured forearms of each patient, no significant differences were revealed. The strength of the grip and pinch showed similar results in both BIN and ESIN groups (Table 2).

Analysis of the functional quantities in the ITT material showed statistically significant differences in the prosupination of the forearm and the dorsal and volar flexion of the wrist. With BIN patients, the prosupination of the forearm was 165° (SD 16°) and with ESIN 148° (SD 24°) (95% CI 1.4 to 31.8, *p* = 0.033). The combined dorsal and volar flexion of the wrist measured 176° (SD 17°) with BIN and 163° (SD 13°) with ESIN (95% CI 1.5 to 23.4, *p* = 0.028).

The mean carrying angle of the upper extremity was 14.9° (SD 4.7°) in the BIN group and 14.4° (SD 5.1°) in the ESIN group (95% CI −3.3 to 4.1, *p* = 0.81). There was no difference in the carrying angles when the fractured arms (14.7°, SD 4.8°) were compared to the uninjured arms (13.6°, SD 7.2°) (95% CI −2.2 to 4.3, *p* = 0.523).

Fourteen out of 15 patients (93%) in the BIN group and 13 out of 15 (87%) in the ESIN group achieved the maximum 100 points in their Mayo elbow performance scores (95% CI of difference −19% to 33%, *p* = 0.999). The mean score for the BIN patients was 99.0 (SD 1.87) and for the ESIN group 98.0 (SD 3.47). The reason for the decrease in the score was mild pain in all three patients who did not achieve full points. The results of QuickDASH were similar in both groups, with the mean score for the BIN group being 0.75% (SD 2.04%) and for the ESIN group 0.61% (SD 1.81%), denoting close to full ability. With the ESIN, there were four (27%) patients who reported pain in the VAS and none in the BIN group (95% CI on difference −8% to 47%, *p* = 0.186), although one patient reported mild pain in their Mayo elbow performance score. The mean value of pain with ESIN was 1.8 mm (range 1–20, SD 4.5 mm).

### 3.2. Degradation of the Biodegradable Implants

**Figure 2:**
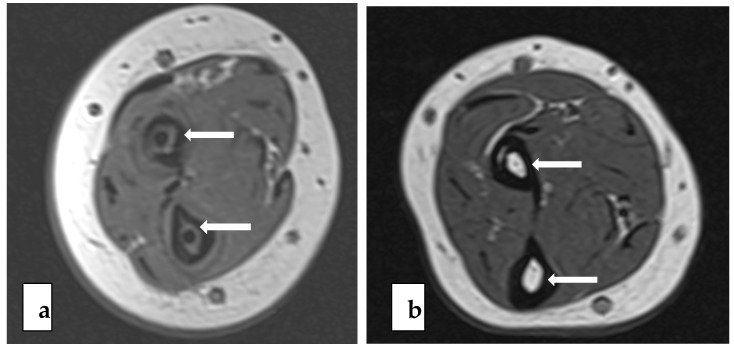
T1-weighted axial images showing low-signal biodegradable nails (white arrows) one month postoperatively (**a**) and faint nail residuals 6.5 years postoperatively (**b**).

**Figure 3:**
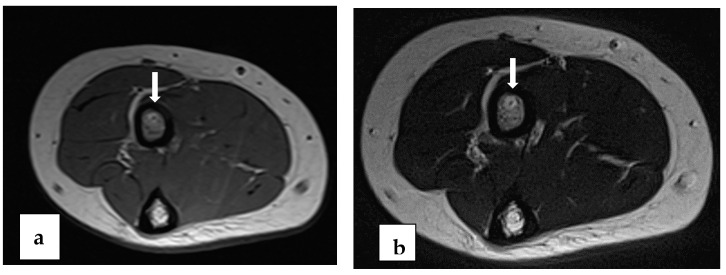
T1-weighted (**a**) and T2-weighted (**b**) axial images with bright halo (white arrow) surrounding a faint residual of BIN in radial bone.

The PLGA implants of the BIN group were completely absorbed in 15 out of 26 bones (58%, 95% CI 37% to 77%) and almost completely absorbed in 11 bones, according to the MR images. The nail residuals appeared as faint, linear, low-signal short markings (Figure 2). The nail canal disappeared completely in 20 (77%, 95% CI 56% to 91%) forearm bones and almost completely in six bones. These residual canals demonstrated higher signals than the cancellous bone in the T1 and T2 images of the MRI. In two out of all 26 bones, thin nail residuals were found to be surrounded by a 3–4 mm halo effect (Figure 3). The tri-calcium-phosphate marker at the end of the nail was visible in 24 out of 26 (92%, 95% CI 76% to 98%) forearm bones in the MR images. (Table 3) The formation of intraosseal cysts was not detected.

### 3.3. Other Imaging Findings

In the forearm MRI of the BIN group, no soft tissue or bone oedema were detected, and fatty bone marrow was intact in all fractured forearm bones. The cortical perforation of the nail entrance was seen in five out of 26 bones (19%, 95% CI 7% to 39%). The IOM between the radius and ulna was visible and intact in all cases. Operation-related low-signal artefacts in the soft tissues were found in 11 out of 15 patients (73%, 95% CI 45% to 92%).

**Figure 4:**
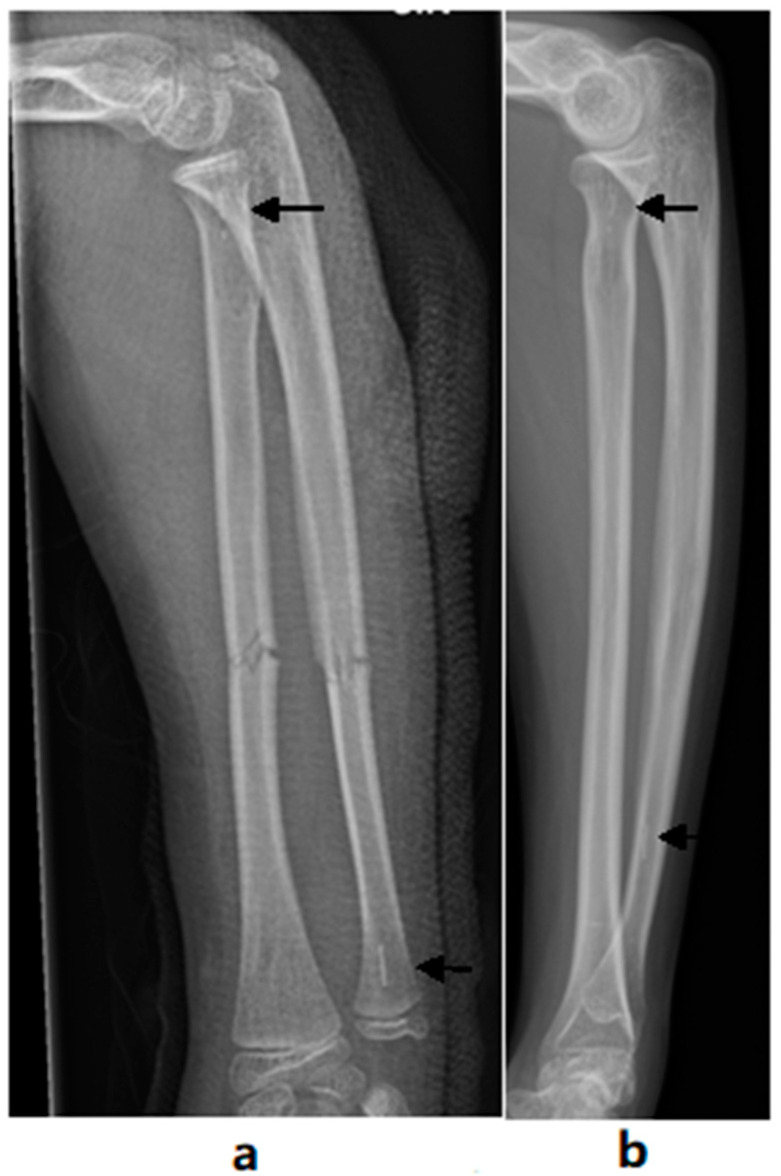
Forearm radiographs with radiolucent β-TCP-tips (black arrows) of the biodegradable nails immediately after operation (**a**) and 6.6 years after operation (**b**).

Clinically significant differences were not found in the alignment of the fractured forearm bones when compared with the non-fractured bones, regardless of fixation material. The lengths of the forearm bones were similar between the fractured and uninjured forearms in both treatment groups. The resorption of the callus was complete in 19 (73%) out of 26 bones in the BIN group and 25 (83%) out of 30 in the ESIN group (95% CI of difference −33% to 12%). The β-TCP tip of the biodegradable nail was visible in the radiographs of all bones from the BIN group (Figure 4).

## 4. Discussion

The gold-standard method of surgical treatment of children’s forearm shaft fractures is intramedullary nailing using flexible intramedullary nails [15,21,22,23,24,25,27,28]. However, there are disadvantages in ESIN; therefore, we developed a new intramedullary fixation method based on biodegrading implants. In this prospective randomized trial with at least four years of follow-up, we found that the long-term clinical outcomes of the study method (BIN) were at least as good as those in the reference method (ESIN). Despite the PLGA study implants being much less stable than the metal alloy implants used in the reference method, they were still stable enough to maintain alignment until bone healing. Due to high elasticity it is assumed to not prevent the remodelling of the fractured forearm bone. In addition, a secondary operation is not needed for implant removal.

Previously, there have been limitations when using biodegradable material in human tissue. Therefore, polymers used in bioabsorbable fixation devises in orthopaedic surgery have been under fast development. Currently, they have special features concerning degradation, with the degradation rate being dependent on the used homopolymers. The study implants used in this research consisted of two polymers that degrade at different speeds: polyglycolide (PGA) and polylactide (PLA). Böstman et al. [31] reported that PGA disappeared from the tissue within nine months (36 weeks) when assessed via a light microscope in an experimental study. Polylevolactic acid (PLLA), which is a stereoisomer of PLA, has a longer degradation time: Pihlajamäki et al. [32] analysed PLLA implants in 10 patients with operatively treated displaced malleolar ankle fractures. At the final follow-up (range of 8 to 52 months postoperatively), all PLLA implants were visible upon MRI and had maintained their size and shape. Poly-L-lactidehydroxyapatite screws in anterior cruciate ligament reconstruction were followed via MRI at 2, 5, and 13 years by Sundarj et al. [33] The bioabsorbable screws had lost their volume at two and five years (MRI imaging) and were completely resorbed at 13 years. Co-polymer PLGA contains both rapidly and slowly degrading polymers. The idea of using a co-polymer was to maintain stability for a sufficiently long time to promote fracture healing while maintaining a reasonable degrading process. Our study, which had at least four years of follow-up, strengthened the conception of co-polymer based implants. The bones showed both good reduction and the fixation material degradation. Previously, non-favourable results of PLGA screws (L-lactic/co-glycolic acid copolymer) were reported by Balestro et al. [10] when they treated recurrent anterior shoulder instability using the Latarjet procedure. Coracoid graft osteolysis was detected in 66.7% (8/12) of the shoulders, and three patients suffered from recurring instability. The PLGA screws were all resorbed within two years when imaged via CT. However, CT and MRI are not comparable methods in assessing the degradation of polymers.

Biodegradable fracture fixation has favourable characteristics in respect to MR imaging, because it does not produce artefacts around the fixation devise.

During the long-term follow-up period, i.e., a mean of 6.8 years after the surgical treatment with biodegradable intramedullary nailing, the fixation material was completely absorbed in more than half of the bones (15/26, 57.7%) and almost completely absorbed in 11 out of 26 (42.3%) bones upon MRI. The remaining small particles of the nails were thin and faint at the long-term follow-up (Figure 2). This is rather different from previous short-term findings at the two-year mark, at which the nails were all visible [30]. Even though the resorption time was longer than previously suspected, the finding supports the use of the study method. In the present study, there were no complications related to the degradation process, such as cysts or osteolysis. However, the finding may encourage further development of the implant material to undergo a more rapid degradation process.

We found that the fatty intramedullary canal had been remodelled at the long-term follow-up. In six bones, some parts of the nail canals were still visible. These residual canals demonstrated higher signal than the cancellous bone in the T1 and T2 images of the MRI. In two out of all 26 bones, thin nail residuals were found to be surrounded by a 3–4 mm halo effect (Figure 3). This is in agreement with the literature, while Pihlajamäki et al. [34] previously reported a rim of a 1–2 mm halo around the PLLA implants that was bright in the T2, PD, and fat-saturated images. The clinical importance of the halo effect is not well understood but it will probably gradually disappear with time upon the continuation of tissue turnover.

A good clinical long-term recovery was found in all patients treated by either BIN or ESIN. One isolated patient with biodegradable nails and two patients with titanium nails reported mild pain in their Mayo elbow performance questionnaires. However, the mean score for the BIN patients was excellent (99.0, SD 1.87) and similar to that of the ESIN patients (98.0, SD 3.47). Similarly, the QuickDASH disability scores were excellent in both groups. The functional recovery, assessed via clinical measurements of range of movements and strength, were equal for both BIN and ESIN, while the pronation of the forearm, flexion of the elbow, and volar flexion of the wrist were statistically significantly better in the BIN group; however, this difference was slight from a clinical point of view. The good long-term outcomes of forearm fractures in this research are in line with the current literature [26,27].

Implant failure occurred in two patients treated with BIN (2/19, 10.5%) vs. none treated with ESIN (0/16) [28]. These patients with re-fractures were teenaged adolescents, a 14-year old boy (67 kg) and a 13-year old girl (40 kg). Their clinical and radiographic results were primarily good. However, they suffered from re-fracture without a new isolated injury at four weeks and the three-month mark postoperatively, respectively. The re-fractures were reduced and stabilized by using a plate and screw fixation. Another two patients, a 10-year old girl and a 9-year old boy suffered from re-fractures related to new high-energy injuries eight months and ten months post-operatively. Their injury mechanisms were falling from a scooter and trampoline jumping. The new fractures of these two patients were treated by using plate and screw fixation.

As a limitation, the removal of the PLGA intramedullary implants would not be straightforward, if needed in case of a new fracture in the same forearm. In this research, two patients, aged 9 and 10, suffered from new high-energy fractures in the previously operated forearms. The removal of the PLGA nails were not attempted; in contrary, open reduction was performed for both and the holes were drilled through the bone cortices and the remaining intramedullary implants, when the new fractures were stabilized with a plate and screws. The need of plate and screw fixation is a disadvantage in that age group, while a repetitive ESIN by replacing the bent intramedullary nails by the intact ones would have been possible after traditional ESIN. However, this disadvantage of BIN must be considered by understanding that ESIN is not complication-free: tendon ruptures, nerve injuries, delayed union, malunion or non-union and re-fractures have been reported as the potential adverse events of ESIN [14,22,24,26,28,35,36].

According to the study plan, all operated extremities were immobilized by using the above-the-elbow cast, regardless on the implants. There is controversial—whether to implement postoperative immobilization with ESIN or not—but immobilization was the current practice in our institutions when the study protocol was designed. Due to lower stability of the BIN implants, we find it clear that postoperative immobilization by casting is an important part of the treatment: the purpose of the cast is in particular to hold alignment, while the intramedullary BIN implants prevents the fracture parts from displacement.

## 5. Limitations of the Study

Although 30 out of 35 (85.7%) of the enrolled patients participated in the follow-up portion of the study at least four years following their operation, the overall number of patients is small. Additionally, during enrolment and operation, the age distribution of the patients was five to 15 years. The bone characteristics of young children are different from those of teenagers, which may affect the support power supplied by intramedullary nails, especially biodegradable nails. A greater sample size would have provided the opportunity to assess the feasibility of biodegradable nails among different age groups.

## 6. Conclusions

After a mean follow-up time of 6.8 years, both BIN and ESIN resulted in excellent clinical outcomes in paediatric forearm shaft fractures. The PLGA biodegradable implants were completely or close to completely resorbed in all patients according to the MRI, and no complications related to the biodegrading process were found. However, two implant failures (2/19, 10.5%) with BIN occurred in teenaged patients shortly after operative treatment vs. none (0/16) with ESIN. Therefore, we find the BIN technique as an option particularly in younger children, but further trials are needed to determine the feasibility of the procedure in older children and teenagers.

## Figures and Tables

**Table 1:** Patient and Injury Characteristics.

		BIN	ESIN
Age (mean, y) *		10.2 ± 1.7	9.9 ± 3.2
Age (mean, y) **		17.2 ± 1.9	17.1 ± 3.2
Gender **			
	Male	5	7
	Female	10	8
Weight *		36.7 ± 11.4	35.0 ± 12.4
Fracture *			
	Both bone	18	15
	Radius only	1	1
	Ulna only	0	0
Mechanism of injury *			
	Fall	9	7
	Trampoline jumping	6	6
	Sports	4	3

*, at enrolment, *n* = 35; **, at four years visit, *n* = 30.

**Table 2:** Functional results of the upper extremity at mean 6.8 years after forearm fracture operation using Biodegradable Nail (BIN) or Elastic Stable Intramedullary Nail (ESIN).

	BIN (*n* = 15)		ESIN (*n* = 15)			
	Mean	SD *	Mean	SD	95% CI ** of the Difference	*p*-Value ***
Supination (°)	86.4	12.7	79.5	17.4	−4.5–18.3	0.228
Pronation (°)	78.5	5.9	72.7	8.0	0.6–11.1	0.030
Flexion (elbow, °)	153.8	7.4	144.8	5.2	4.2–13.8	0.001
Extension (elbow, °)	−8.1	5.9	−8.3	6.2	−4.2–4.8	0.905
Dorsiflexion (wrist, °)	86.9	14.6	80.2	9.8	−2.6–16.0	0.153
Volar flexion (wrist, °)	88.9	6.0	82.7	7.9	0.9–11.5	0.023
Grip (Nm)	29.6	10.9	31.7	14.4	−11.6–7.5	0.666
Pinch (Nm, *n* = 14)	6.6	2.7	5.9	2.4	−1.3–2.7	0.469

* SD = standard deviation; ** CI = confidence interval; *** *p*-value measured by using independent *t*-tests.

**Table 3:** Imaging findings of biodegradable nails in MR.

	*n* (26)	%
Absorption of the nail		
Complete	15	58
Almost complete	11	42
Disappearance of the nail canal		
Complete	20	77
Almost complete	6	23
Visible TCP-tip	24	92
Non-visible TCP-tip	2	8
Continuous fatty bone marrow	26	100

## Data Availability

The data presented in this study are available on request from the corresponding author. The data are not publicly available due to privacy reasons.
